# Intrapancreatic accessory spleen false positive to 68Ga-Dotatoc: case report and literature review

**DOI:** 10.1186/s12957-019-1660-2

**Published:** 2019-07-09

**Authors:** Francesco Lancellotti, Luca Sacco, Saverio Cerasari, Vittoria Bellato, Simone Cicconi, Antonio Ciardi, Edoardo Maria Muttillo, Tiziana Feola, Roberto Caronna, Piero Chirletti

**Affiliations:** 1 Department of Surgical Sciences, Sapienza University of Rome, Viale del Policlinico 155, 00161 Rome, Italy; 2 Department of Radiological Oncological and Pathological Sciences, Sapienza University of Rome, Viale del Policlinico 155, 00161 Rome, Italy; 3 Department of Experimental Medicine, Sapienza University of Rome, Viale del Policlinico 155, 00161 Rome, Italy

**Keywords:** Intrapancreatic accessory spleen, False positive to 68Ga-Dotatoc, Neuroendocrine tumor, Pancreatic mass

## Abstract

**Background:**

Intrapancreatic accessory spleen (IPAS) is an uncommon finding of pancreatic mass. Differential diagnosis with pancreatic tumor, especially with non-functional neuroendocrine tumor (NF-NET), may be very hard and sometimes it entails unnecessary surgery. A combination of CT scan, MRI, and nuclear medicine can confirm the diagnosis of IPAS. 68-Ga-Dotatoc PET/CT is the gold standard in NET diagnosis and it can allow to distinguish between IPAS and NET.

**Case presentation:**

A 69-year-old man was admitted to our hospital for an incidental nodule in the tail of the pancreas with focal uptake of 68-Ga-dotatate at PET/CT. NET was suspected and open distal splenopancreatectomy was performed. Pathologic examination revealed an IPAS.

**Conclusion:**

This is the second IPAS case in which a positive 68Ga-Dotatoc uptake led to a false diagnosis of pancreatic NET. Here is a proposal of a literature review.

## Background

Accessory spleen is a congenital abnormality consisting of normal splenic tissue in ectopic sites. It arises as a failure of fusion between some of the multiple buds of splenic tissue in the dorsal mesogastrium during embryologic life. This ectopic tissue can be found, in order of frequency in the following: splenic hilum (80%), pancreatic tail (20%), stomach, bowel, and genitals [1, 2]. In autoptic studies, the accessory spleen has an incidence of 10% and IPAS of 2% [3], but clinical incidence, despite rare, is growing probably related to the improvement of diagnostic imaging accuracy. Rarely a specific abdominal pain or idiopathic thrombocytopenic purpura (not responsive to splenectomy) could be present in patients with IPAS [4]. Because asymptomatic, IPAS is almost always found incidentally as an undefined pancreatic mass similar to NETs [5]. The frequency of functional pancreatic NETs (F-P-NETs), similar to that of non-functional pancreatic NETs (NF-P-NETs), is probably increasing [5] due to the widespread use of high-quality imaging techniques [6]. Recent studies have shown imaging with 68-Ga-labeled somatostatin analogs with PET/CT to be highly sensitive and specific for P-NETs [7]. We are illustrating a 69-year-old man with a mass in the tail of the pancreas found during his follow-up for previous sigmoid colon adenocarcinoma. Because of a positive result at 68-Ga-Dotatoc PET/CT and volumetric increasing of mass during follow-up, a P-NET was suspected.

## Case presentation

A 69-year-old man with a past medical history significant for hypertension and sigmoid adenocarcinoma (UICC 2012: pT4 pN2 M0) underwent sigmoid colectomy and adjuvant chemotherapy (2015). No pancreatic mass was described at the computed tomography (CT) images performed after colectomy. During the follow-up, a magnetic resonance imaging (MRI) showed a 1.5-cm nodule of the pancreatic tail, non-homogeneous, hyperintense on T2-weighted and hypointense on T1-weighted sequence, and a focal lesion inside hyperintense on T1. Endoscopic ultrasonography (EUS) revealed a 1.5-cm anechoic nodule, with two hyperechoic foci ascribable to calcifications. The needle biopsy (EUS-FNA) was not performed due to difficulties of endoscopic examination (the nodule was too far from the duodenal wall). 18-Fluorodeoxyglucose positron emission tomography ([18-F] FDG-PET) did not show FDG uptake.

**Fig. 1:**
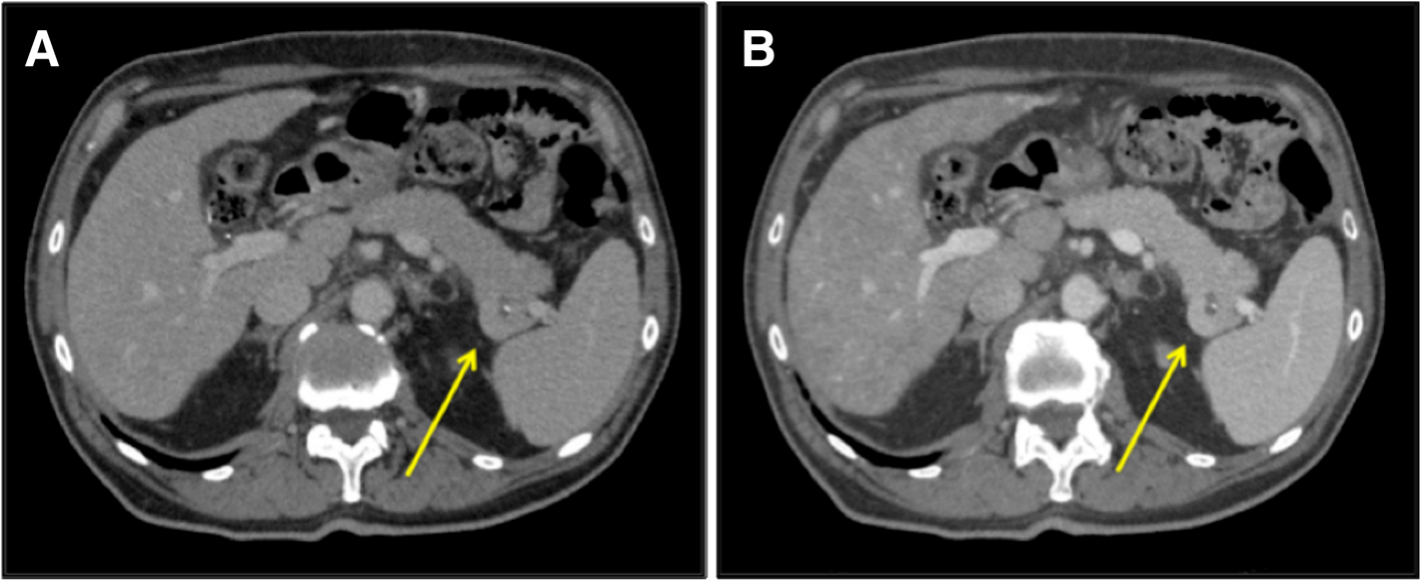
Incidental mass in the tail of the pancreas (arrow). CT revealed a mass well delimited with homogeneous contrast enhancement. **a** Venous and **b** arterial phase axial CT images

**Fig. 2:**
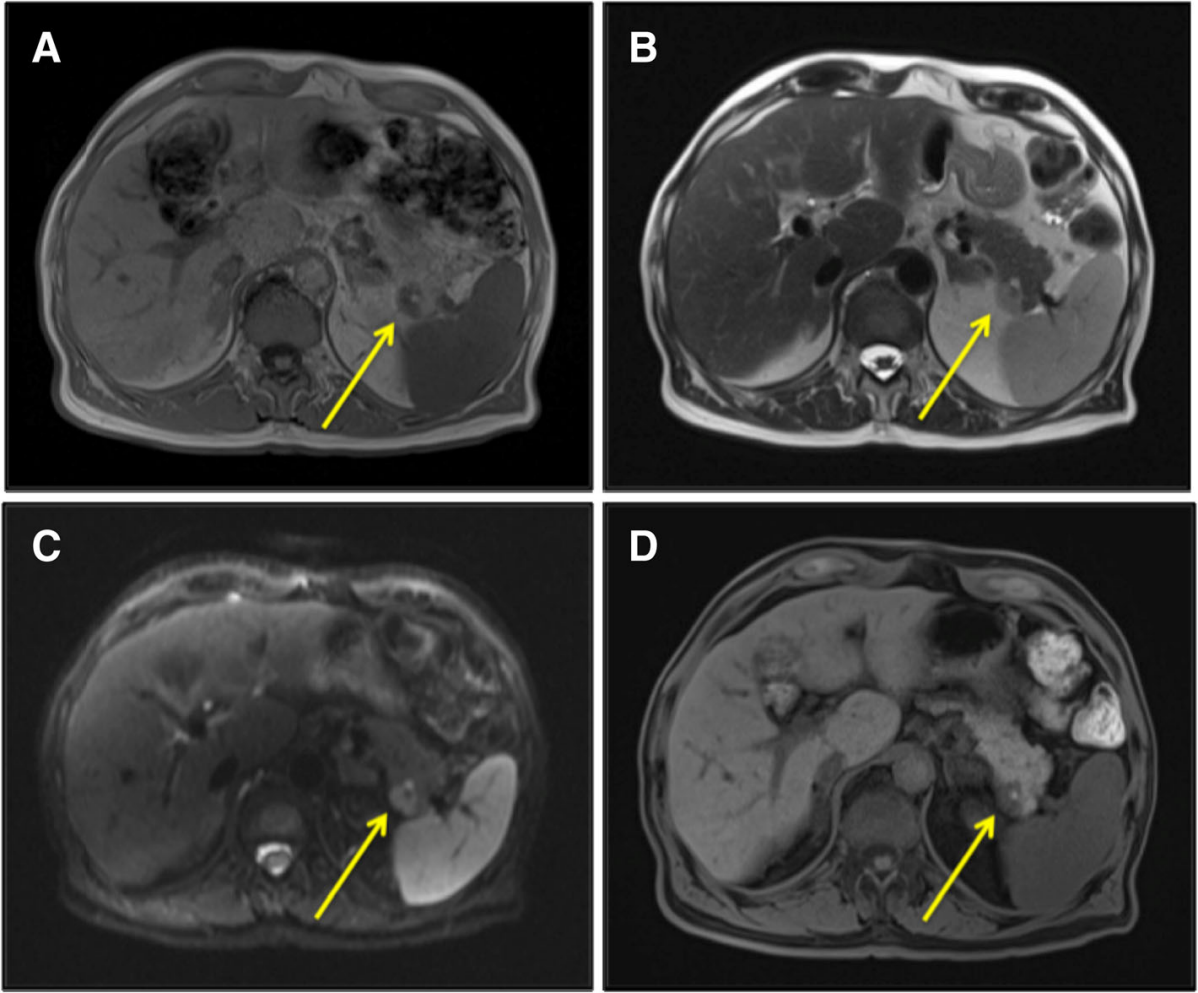
MRI confirmed a 2-cm nodule of the tail of the pancreas hypointense on T1 (**a**) and hyperintense on T2 (**b**)-weighted sequence with a focal lesion inside hyperintense on T1. Diffusion-weighted MR imaging shows a high restriction (**c**) and hypointense in T1 fat sat sequence (**d**)

**Fig. 3:**
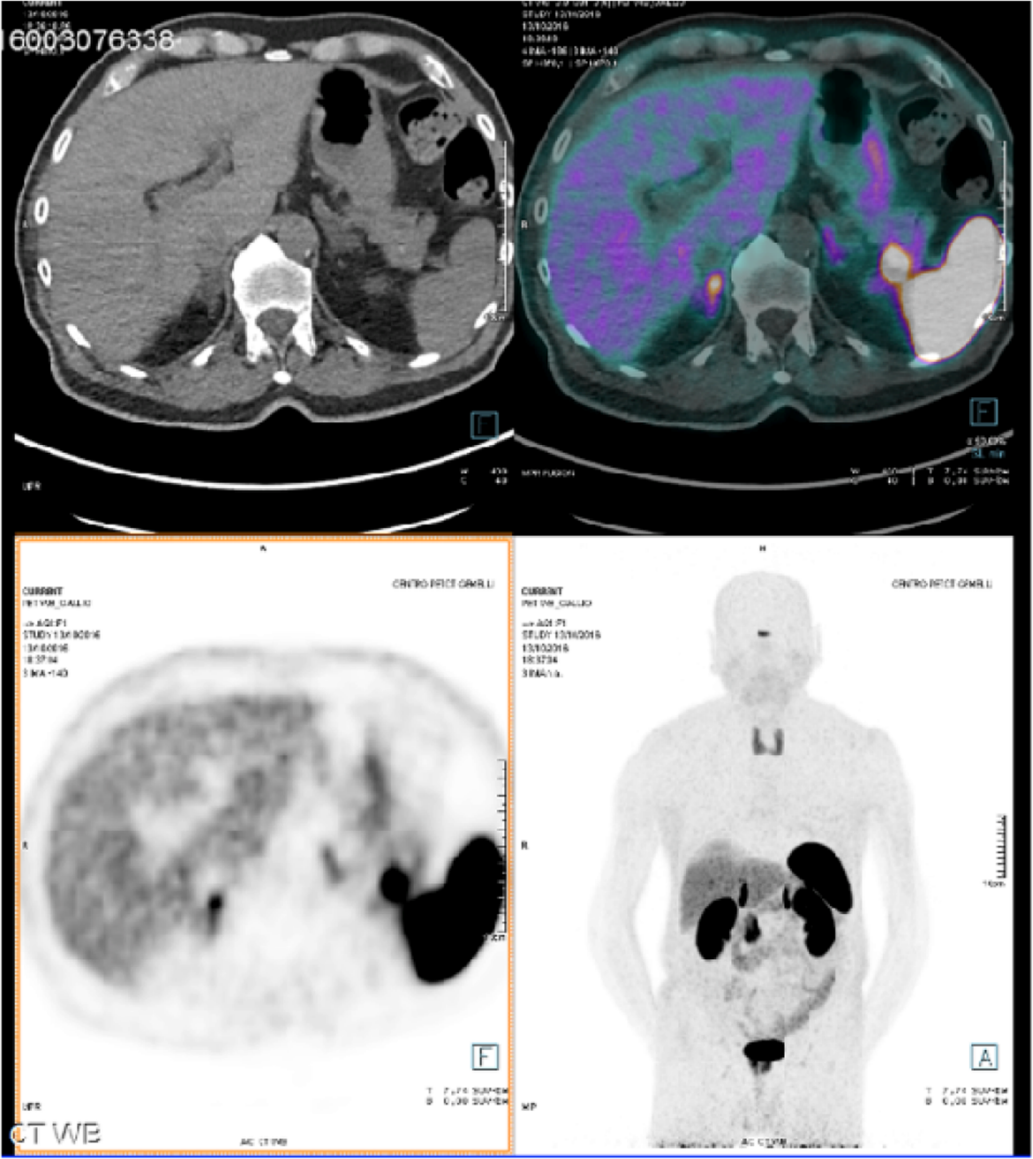
The PET image shows an indeterminate pancreatic tail nodule with enhanced uptake of 68-Ga-dotatate at PET-CT fusion image (false positive)

**Fig. 4:**
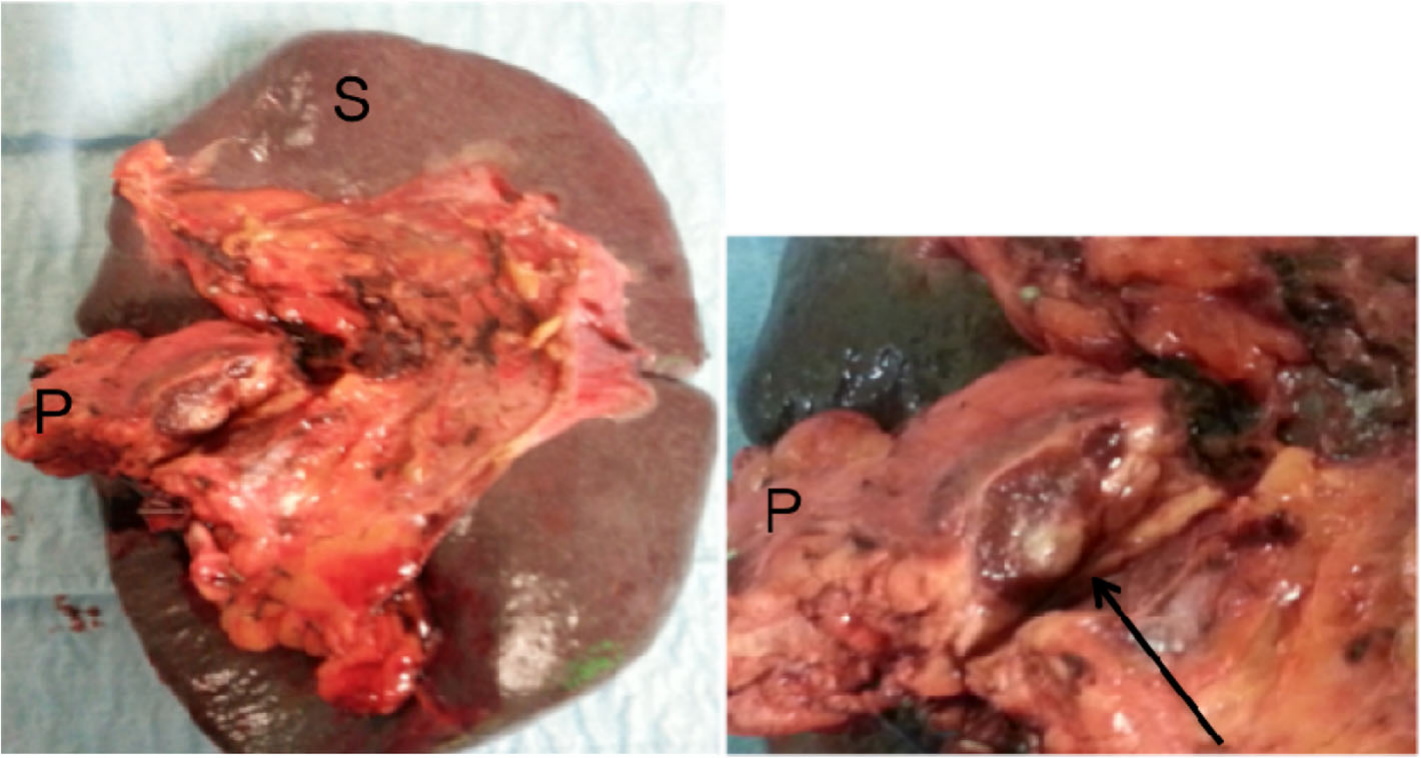
Gross pathologic findings of IPAS: S the main spleen; P the tail of the pancreas. The arrow shows a reddish nodule with epidermoid cyst surrounded by pancreatic parenchyma

Follow-up indication was given. After 8 months, this lesion showed a volumetric increase (2 cm) both at computed tomography (CT) and MRI (Fig. 1 and Fig. 2) without pathological uptake at FDG-PET. Tumoral markers (CA 19.9, CEA, alpha-fetoprotein) were negative. A 68-Ga-Dotatoc PET/CT, more sensitive and specific for neuroendocrine tumor (NET), showed a focal area of uptake (Fig. 3), but neuroendocrine markers (gastrin, chromogranin A, calcitonin, 5-hydroxytryptophan) were negative. Given the suspicion of NF-P-NET and close contact to splenic vessels, the patient underwent open distal splenopancreatectomy (Fig. 4) with an unremarkable postoperative course.

**Fig. 5:**
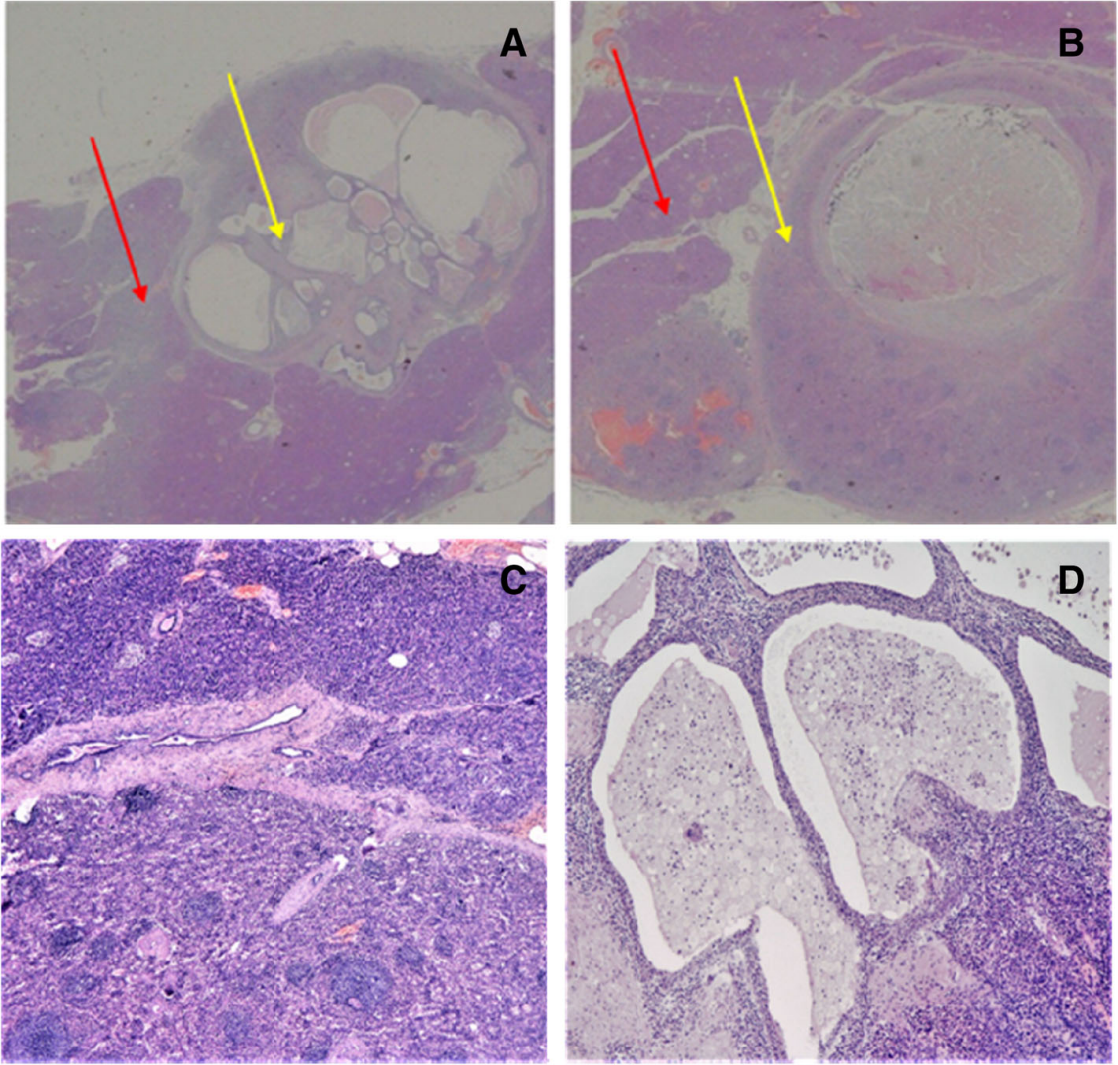
Microscopic findings (hematoxylin and eosin staining). **a**, **b** It is possible to observe the interface between pancreatic parenchyma (red arrow) and accessory spleen with epidermoid cyst (yellow arrow) (H&E, × 4). **c** Histological picture of intrapancreatic splenic parenchyma with adjacent normal pancreas (H&E, × 40). **d** Multiseptated intrasplenic epithelial cyst, with multilayered squamous epithelium (H&E, × 40)

Definitive histologic examination revealed an intrapancreatic accessory spleen, with multiseptated epidermoid cyst (Fig. 5).

## Discussion

In the English literature, there are, up to date, only 144 articles of which 115 are case reports concerning diagnosis and treatment of IPAS. From this data review, it was observed that most patients with IPAS underwent surgery without a preoperative definitive diagnosis and that IPAS was often put in differential diagnosis with NET or rarely with adenocarcinoma or metastatic tumors [8]. In Table 1, we report the literature results of diagnostic procedures performed, when clearly specified, in patients with IPAS [9–31]. Despite the high diagnostic accuracy of morphological and scintigraphic exams, IPAS was often unidentified and unnecessary surgery was performed in 66.6% of patients. False-negative results have been reported also after endoscopic ultrasonography-guided fine needle aspiration biopsy (EUS-FNA) and after Tc-99m heat-damaged red blood cell (HDRBD) scintigraphy.

**Table 1:** Literature review of 87 cases of IPAS confirmed by postoperative histological examination or by follow-up: diagnostic hypothesis and related surgical treatment [9–31]. Fifty-eight cases treated with surgery (66.6%)

	Patients, *n*	Diagnostic hypothesis						Unnecessary surgery	
		IPAS	U	NET	Other	Misleading, *n* (%)		*n*	%
CT scan	57	13	16	24	4	44	(77.1)	31	54.3
MRI	60	32	10	11	7	28	(46.6)	40	66.6
EUS	30	3	14	12	1	27	(90)	15	50
EUS-FNA	20	15	2	3	0	5	(25)	8	40
Octreoscan	9	0	7	2	0	2	(22.2)	7	77.7
HDRBD	10	6	0	4	0	4	(40)	6	60

*U* unclear

The context is usually a CT/MRI incidental diagnosis of a nodule localized in the pancreatic tail, between 1 and 3 cm, well-delimited, homogeneous, and hypervascular: IPAS should be suspected and more examinations required.

Tumoral and neuroendocrine markers have a limited role in the differential diagnosis. In pancreatic carcinoma, the most important tumoral markers are CA 19.9, CEA, CA125 (sensitivity of 81.3%, 39%, and 56.4%; specificity of 75.9%, 91.4%, and 77.6% respectively) [32, 33]. Approximately 50% of NET are non-functional tumors; in functional ones, neuroendocrine marker sensitivity is about 80% while specificity is much lower [34]. However, false-positive cases consisting of IPAS with increased tumoral and neuroendocrine markers have been reported [10, 13]. Therefore, serological markers are not useful in the differential diagnosis between IPAS and NET or pancreatic carcinoma, as in our case.

For these reasons, a careful evaluation of the radiological images is very important.

At CT, IPAS shows attenuation and enhancement similar to the spleen in all phases, more than pancreatic parenchyma. Pancreatic tumors, instead, show a greater attenuation in the arterial phase and less in the venous phase [13, 19].

At MRI, IPAS often shows a low signal intensity in T1 and high intensity in T2 if compared to pancreatic parenchyma; moreover, intratumoral hemorrhage and necrosis are absent in IPAS [19]. The key to suspect IPAS at MRI is detecting the signal intensity of the mass similar to the spleen in all sequences [13]. On the other hand, Kim et al. demonstrated that sometimes IPAS signal intensity is slightly brighter than the spleen on the T2-weighted images, and this finding is due to the higher white-to-red pulp ratio of IPAS [13]. Recently, Jang et al. [23], in a retrospective study, have considered diffusion-weighted MRI (RMDW) value in the differential diagnosis between IPAS and solid pancreatic tumors of less than 3 cm showing a 90% sensitivity and specificity. An Italian group in 2005 correctly diagnosed IPAS (without surgery), observing the same pattern of the spleen at CT and at MRI with reticuloendothelial system-specific contrast medium (ferucarbotran), confirmed by mass stability after 16 months of follow-up [25].

Nuclear medicine has surely a role in IPAS diagnosis. Octreoscan and 68-Ga-PET/CT have been considered reliable in case of NET, while Tc-99m heat-damaged red blood cell (HDRBD) is specific for splenic tissue. According to variable expression NET somatostatin receptors (high or low), it is possible unfortunately unidentify these tumors only with radiolabelled somatostatin analog agent [35]. Furthermore, there are non-tumoral processes (autoimmune diseases, pneumonias, etc.) and some tissues (spleen, kidney, thyroid, liver) that can have avidity for scintigraphy contrast, causing false positives. Octreoscan sensitivity and specificity for NET is about 80% while 68-Ga-Dotatoc seems to be superior (sensitivity and specificity range 80–100%) [36]. Therefore, for NET diagnosis, the actual gold standard is 68-Ga-PET-CT. To date, in the English literature, there is only one IPAS case in which a positive 68-Ga-PET/Tc uptake led to a wrong diagnosis of pancreatic NET [1] probably due to physiological radioisotope uptake in splenic tissue (false positive) [30, 37, 38]. We present the second case of IPAS positive at 68-Ga-PET/CT. About 68-Ga-PET-CT sensitivity and specificity, it should be considered that the splenic tissue uptake of 68-Ga-Dotatoc is highly variable [39, 40]. The accuracy seems significantly higher for NET restaging than diagnosis [41] and then a histological confirmation for final diagnosis could be still considered [42]. Therefore, the diagnostic reliability of the 68-Ga-PET/TC in terms of differential diagnosis between NET and IPAS is still debated.

Technetium 99-m HDRBD scintigraphy is a specific technique for splenic tissue identification because of the physiological radioisotope uptake: focal uptake in the pancreatic tail suggests the presence of splenic intrapancreatic tissue [17]. Method limits are inferior spatial resolution compared to other cross-sectional imaging modalities and need for a certain quantity of spleen functional ectopic tissue to visualize marked cells (cutoff dimension of 1.1 cm according to Kim et al.) [13]. Consequently, Tc-99m HDRBC scintigraphy is an exam that can be used as a confirmatory test for IPAS (positive predictive value) but false-negative results have been reported [19, 23, 31] (Table 1). In our case, it was not performed because 68-Ga-PET/TC positive result was considered conclusive.

EUS-FNA, despite a certain rate of complications and false negatives, gives significant morphological and cytological informations. Schreiner et al. in 2008 first described a series of 3 IPAS identified by EUS, suggesting the need for a histologic diagnosis (FNA biopsy) in case of unconclusive imaging [14]. Cytological features for differential diagnosis of IPAS from other pancreatic nodules were described by Tatsas et al. [22]. In case of IPAS, there is a population of inflammatory cells (principally lymphocytes, but also monocytes, neutrophils, eosinophils) while immunocytochemical staining of CD8 specifically highlights endothelial cells of the thin-walled blood vessels [22]. EUS-FNA biopsy has a high (80–90%) sensitivity and specificity for pancreatic neuroendocrine tumors although its accuracy for such tumors is considered lower than the accuracy for pancreatic adenocarcinoma [21]. Although false-positive results have been reported [11, 16, 31], the rate of unnecessary surgery after EUS-FNA biopsy is lower (40%) compared with other morphological exams (Table 1). There is also the possibility of performing a contrast-enhancement EUS (CEUS) with intravenous contrast (Levovist or Sonazoid) without FNA: it may become the gold standard in IPAS diagnosis [43].

Finally, there is another method proposed for IPAS diagnosis: confocal laser endomicroscopy (CLE) [24]. CLE is an endoscopic technique which allows to obtain the mucosal images with a magnification of about × 1000. This technique allows to identify cellular and subcellular microstructures and an in vivo histological diagnosis (optical virtual biopsy). The use of needle-based probe CLE prior to EUS-FNA in the diagnosis of pancreatic masses may increase diagnostic accuracy [24].

To date, there are no guidelines but recommended diagnostic algorithms to suspect and diagnose IPAS have been proposed by Spencer et al., Li et al., and Baugh et al. [19, 31, 44].

## Conclusion

In the differential diagnosis of solid nodules in the pancreatic tail, IPAS should always be considered. Besides increasing clinical incidence due to the improvement of imaging quality, it remains a non-neoplastic lesion with no surgical indication. IPAS should be suspected in the presence of some features: incidentally asymptomatic lesion, localization in the tail of the pancreas, dimension between 1 and 3 cm, well-delimited homogeneous and hypervascular nodule, similar attenuation to the spleen on CT and MRI, negativity of neuroendocrine, and tumoral markers. A combination of CT, MRI, nuclear medicine examinations, and EUS-FNA biopsy could be necessary for a diagnosis of IPAS because none of them are individually conclusive. False positives or false negatives are possible, as in our clinical case with 68-Ga-Dotatoc PET/CT false positive.

## Data Availability

All data generated or analyzed during this study are included in this published article.

## Abbreviations

[18-F] FDG-PET: 18-Fluorodeoxyglucose positron emission tomography
CEUS: Contrast-enhancement EUS
CLE: Confocal laser endomicroscopy
CT: Computed tomography
EUS: Endoscopic ultrasonography
EUS-FNA: Endoscopic ultrasonography-guided fine needle aspiration biopsy
HDRBD: Tc-99m heat-damaged red blood cell
IPAS: Intrapancreatic accessory spleen
MRI: Magnetic resonance imaging
NF-NET: Non-functional neuroendocrine tumor
